# Extracellular Matrix Mineralization Promotes E11/gp38 Glycoprotein Expression and Drives Osteocytic Differentiation

**DOI:** 10.1371/journal.pone.0036786

**Published:** 2012-05-07

**Authors:** Matthew Prideaux, Nigel Loveridge, Andrew A. Pitsillides, Colin Farquharson

**Affiliations:** 1 Division of Developmental Biology, The Roslin Institute, The Royal (Dick) School of Veterinary Studies, University of Edinburgh, Edinburgh, United Kingdom; 2 Department of Veterinary Basic Sciences, Royal Veterinary College, London, United Kingdom; 3 Bone and Orthopaedic Research Groups, University of Cambridge, Cambridge, United Kingdom; INSERM U791, LIOAD, France

## Abstract

Osteocytes are terminally differentiated osteoblasts which reside in a mineralized extracellular matrix (ECM). The factors that regulate this differentiation process are unknown. We have investigated whether ECM mineralization could promote osteocyte formation. To do this we have utilised MLO-A5 pre-osteocyte-like cells and western blotting and comparative RT-PCR to examine whether the expression of osteocyte-selective markers is elevated concurrently with the onset of ECM mineralization. Secondly, if mineralization of the ECM is indeed a driver of osteocyte formation, we reasoned that impairment of ECM mineralization would result in a reversible inhibition of osteocyte formation. Supplementation of MLO-A5 cell cultures with ascorbic acid and phosphate promoted progressive ECM mineralization as well as temporally associated increases in expression of the osteocyte-selective markers, E11/gp38 glycoprotein and sclerostin. Consistent with a primary role for ECM mineralization in osteocyte formation, we also found that inhibition of ECM mineralization, by omitting phosphate or adding sodium pyrophosphate, a recognized inhibitor of hydroxyapatite formation, resulted in a 15-fold decrease in mineral deposition that was closely accompanied by lower expression of E11 and other osteocyte markers such as *Dmp1*, *Cd44* and *Sost* whilst expression of osteoblast markers *Ocn* and *Col1a* increased. To rule out the possibility that such restriction of ECM mineralization may produce an irreversible modification in osteoblast behaviour to limit E11 expression and osteocytogenesis, we also measured the capacity of MLO-A5 cells to re-enter the osteocyte differentiation programme. We found that the mineralisation process was re-initiated and closely allied to increased expression of E11 protein after re-administration of phosphate or omission of sodium pyrophosphate, indicating an ECM mineralization-induced restoration in osteocyte formation. These results emphasise the importance of cell-ECM interactions in regulating osteoblast behaviour and, more importantly, suggest that ECM mineralization exerts pivotal control during terminal osteoblast differentiation and acquisition of the osteocyte phenotype.

## Introduction

Osteocytes are the most numerous cell type in bone. One of their defining characteristics is the formation of multicellular networks permeating the mineralized extracellular matrix (ECM), through which they are believed to play pivotal mechanomodulatory roles in directing bone formation and bone resorption in response to load-bearing [1]. Despite this crucial contribution to bone remodelling, the mechanisms controlling osteocyte formation from their osteoblast precursors remain largely undefined.

The process during which an osteoblast differentiates into an osteocyte, termed osteocytogenesis, involves transition from the cuboidal-like osteoblastic morphology to a dendritic shape characteristic of an osteocyte, as it becomes embedded within the bone ECM [2]. This is accompanied by a loss of cell volume, reduced organelle content and the formation and elongation of thin cytoplasmic projections. These projections pervade the bone and, via the canaliculi, connect each osteocyte to its neighbours within the bone ECM and to osteoblasts on the bone surface [2], [3].

This transition from osteoblast to osteocyte is commonly described to be a passive process, during which an osteoblast destined for osteocytogenesis slows ECM production and becomes surrounded by the osteoid synthesised by its neighbours [3]–[5]. Studies by Holmbeck *et al.* [6], however, show that cleavage of collagen by MT1-MMP (membrane type 1 matrix metalloproteinase), a membrane associated member of the MMP family, is required for the development of osteocyte projections. Thus, osteocytogenesis may be an active process involving activation of key pathways in specific osteoblasts to induce osteocytic differentiation.

Many factors show altered expression during osteocytogenesis [7]. Some factors such as SOST/sclerostin, which is specific to osteocytes [8], [9], regulate bone formation and inhibit osteoblast differentiation by antagonising the Wnt-signalling pathway [10], [11]. Others, such as *Phex* (phosphate-regulating gene with homologies to endopeptidases on the X chromosome) and *Dmp1* (dentin matrix protein 1) have been shown to be involved in phosphate homeostasis [12] and ECM mineralization [13], [14], another key feature of the osteoblast-osteocyte transition.

The E11/gp38 transmembrane glycoprotein is the earliest osteocyte specific protein shown to be expressed during osteoblast-to-osteocyte transition and therefore may be considered as an ideal driver of this osteocytogenic process [15]. E11 is present in osteocytes in the newly formed matrix (osteoid) as well as those in the mineralized bone where its expression is increased in response to fluid-flow shear stress [15], [16]. Furthermore, siRNA E11 knockdown abrogates the elongation of osteocyte processes in response to fluid-flow sheer stress [15]. E11 is also expressed in kidney and lung where it is alternatively known as podoplanin and T1alpha/RT140, respectively [17]–[19]. By binding to members of the ERM (ezrin, radixin, and moesin) family of proteins [18]–[20], E11 is implicated in cytoskeletal re-arrangement and cell migration. The cell surface glycoprotein CD44 is also expressed by osteocytes, but not osteoblasts, *in vivo* [21]. Physical association of CD44 and E11 has been observed in tumour vascular endothelial cells [22] and squamous stratified epithelial cells [23] and similar interactions may occur in osteocytes to influence cell morphology.

The extracellular environment, and in particular the ECM within which cells become entombed, is also considered to exert a vital influence during osteocytogenesis. Previous observations suggest that the local ECM becomes mineralized concomitantly with osteoblast-osteocyte differentiation [3], [24] and studies by Irie *et al.* [25] have shown that ECM mineralization is important for maturation of immature rat osteocytes. These *in vivo* studies demonstrated restricted formation of osteocyte dendritic projections and reduced sclerostin expression in newly formed, non-mineralized ECM of bisphosphonate-treated rats [25]. ECM mineralization occurs when inorganic phosphate and calcium crystals (which together form calcium-phosphate complexes and finally bone hydroxyapatite: the inorganic component of bone) are deposited along the type-I collagen fibrils, which comprise the ECM [26]. Thus, whilst it is well established that osteocytes within this mineralized bone differ greatly in both morphology and gene and protein expression compared to osteoblasts on the bone surface from which they originated, the influence of ECM mineralization on osteocyte differentiation remains to be fully elucidated.

In this study we have used the murine MLO-A5 cell line, which both synthesizes a mineralized matrix and expresses osteocyte markers [17], [27] to examine the role of matrix mineralization during osteocytogenic transition. Our studies indicate the importance of ECM-cell interactions and the crucial role of ECM mineralization as an essential regulator of osteocytogenesis.

## Methods

### MLO-A5 cell culture

The MLO-A5 murine pre-osteocyte cell line was obtained from Lynda Bonewald (University of Missouri-Kansas City, USA). Cells were plated at 35×10^3^ cells/cm^2^ in multi-well plates and cultured in α-MEM medium supplemented with 5% (v/v) FBS, 5% (v/v) calf serum (CS) (Invitrogen, Paisley UK) and 50 µg/ml gentamicin (Invitrogen) (‘culture medium’) at 37°C with 5% CO_2_. At confluency (day 0), the medium was changed to α-MEM supplemented with 10% FBS, 50 µg/ml gentamicin, 5 mM β-glycerol 2-phosphate disodium salt hydrate (βGP, Sigma UK) and 100 µg/ml ascorbic acid (AA, Sigma) in order to promote differentiation and matrix mineralization (‘differentiation medium’). The MLO-A5 cells were incubated at 37°C in a humidified atmosphere containing 5% CO_2_ and the medium was changed every 3 days for a further 15 days. Individual cultures were collected at 3-day intervals for Alizarin red staining for the assessment of matrix mineralization and for RNA and protein extraction.

### Primary calvarial osteoblast culture

Primary calvarial cells were isolated from 3 day-old C57Bl/6 mice as described in [28]. Briefly, excised calvaria were digested with 1 mg/ml collagenase type II for 10 minutes to remove fibroblasts and marrow cells. Calvaria were then digested with 1 mg/ml collagenase (30 minutes), 4 mM EDTA (10 minutes) and 1 mg/ml collagenase (30 minutes). The cells were collected from each digest, resuspended in α-MEM supplemented with 10% FBS and 50 µg/ml gentamicin and cultured at 37°C with 5% CO_2_ until confluent. For experiments, cells were seeded at a density of 1.5×10^4^ cells/cm^2^. At confluency (day 0), growth medium was supplemented with 2.5 mM ßGP and 50 µg/ml AA for up to 28 days to induce ECM mineralization. The medium was changed every second/third day.

### Inhibition of MLO-A5 matrix mineralization

MLO-A5 cells were cultured as described and upon reaching confluency the medium was changed to differentiation medium lacking βGP (AA alone) or complete differentiation medium supplemented with 500 µM sodium pyrophosphate (PP_i_) (Sigma); found previously to be required in order to achieve full inhibition of mineralization in MLO-A5 cell cultures (data not shown). Medium was changed every 3 days and individual cultures were collected at defined time points (see results). As a restriction of matrix mineralization may irreversibly modify osteoblast behaviour to limit osteocytogenesis, the capacity of MLO-A5 cells to re-enter the osteocyte differentiation programme was also measured, by first restricting and then later promoting ECM mineralization by modifying the medium. PP_i_ was omitted from the fresh medium which was added on day 6, or the cultures that had been treated with medium lacking βGP were later replenished with medium supplemented with βGP. The cells were then grown as previously described for a further 9 days before analysis.

### Assessment and quantification of matrix mineralization

Briefly, cells were washed with phosphate-buffered saline (PBS), and then fixed with 4% paraformaldehyde (PFA) for 5 min at 4°C. After several washes in PBS, cell layers were stained with aqueous 2% (w/v) Alizarin red solution (Sigma) at pH 4.2, for 5 minutes at room temperature, before washing with water, to remove any unbound stain. A photomicrographic image of each culture well was captured to record the distribution of mineral staining, the bound stain subsequently solubilized in 10% cetylpyridinium chloride (Sigma) and the optical density of the resultant solution determined at 570 nm by spectrophotometry (Thermo Multiskan Ascent).

### RNA Extraction and Comparative Real-time PCR

Total RNA was extracted from MLO-A5 cells using Tri-Reagent (Ambion, Huntingdon UK) according to the manufacturer's instructions. After extraction, RNA was precipitated with isopropanol, washed in ethanol and then resuspended in nuclease-free water. Purified RNA was treated with DNase (Ambion) and stored at −80°C. RNA samples (and controls consisting of RNase-free water) were reverse-transcribed into cDNA using Superscipt II reverse transcriptase (Invitrogen) according to the manufacturer's instructions. Real-time PCR (RT-qPCR) was carried out in a Stratagene Mx3000P cycler and each reaction contained 50 ng template DNA, 250 nM forward and reverse primers (Table 1) and 1xFastStart Universal SYBR Green Master Mix (Roche, East Sussex UK). Samples were amplified using the following program; an initial step of 50°C for 2 minutes and 95°C for 2 minutes (1 cycle) followed by 95°C for 15 seconds and 60°C for 30 seconds (40 cycles) and a final step of 95°C for 1 minute, 60°C for 30 seconds, 95°C for 15 seconds and 25°C for 30 seconds (1 cycle). The Ct values for the samples were normalised to that of *Gapdh*, which was determined to be the most stable housekeeping gene for these experiments (data not shown), and the relative expression was calculated using the ^ΔΔ^Ct method [29]. The amplification efficiencies of all the primers were between 90–100%.

**Table 1:** Primer sequences used for qRT-PCR.

Gene	Source	Sequence (5′-3′)
*Phex* (forward)	PrimerDesign	CTAACCACCACTCCCACTT
*Phex* (reverse)	PrimerDesign	CCAATAGACTCCAACCTGAAGA
*E11* (forward)	PrimerDesign	AACAAGTCACCCCAATAGAGATAAT
*E11* (reverse)	PrimerDesign	CTAACAAGACGCCAACTATGATTC
*Cd44* (forward)	PrimerDesign	ATTGGATATGGTCTTGGTTTGGTA
*Cd44* (reverse)	PrimerDesign	TGCCTCTTGGGTGGTGTTT
*Akp2* (forward)	PrimerDesign	GGGACGAATCTCAGGGTACA
*Akp2* (reverse)	PrimerDesign	AGTAACTGGGGTCTCTCTCTTT
*Ocn* (forward)	PrimerDesign	CCGGGAGCAGTGTGAGCTTA
*Ocn* (reverse)	PrimerDesign	TAGATGGGTTTGTAGGCGGTC
*Col1α* (forward)	PrimerDesign	ACCTCACAGATGCCAAGCC
*Col1α* (reverse)	PrimerDesign	ATCTGGGCTGGGGACTGAG
*Gapdh* (forward)	PrimerDesign	Not disclosed
*Gapdh* (reverse)	PrimerDesign	Not disclosed
*Dmp1* (forward)	Ambion	Not disclosed
*Dmp1* (reverse)	Ambion	Not disclosed
*Sost* (forward)	Ambion	Not disclosed
*Sost* (reverse)	Ambion	Not disclosed

### Western Blotting

Protein lysates were extracted from MLO-A5 cells in RIPA buffer (150 mM NaCl, 1.0% IGEPAL® CA-630, 0.5% sodium deoxycholate, 0.1% SDS, 50 mM Tris, pH 8.0) (Sigma) containing protease inhibitors (Roche). Protein concentrations were determined using the DC assay (Bio-Rad, Hemel Hempsted, UK) and 15 µg of protein was separated using a 10% bis-tris gel and then transferred to a nitrocellulose membrane and probed with goat anti-mouse E11 (1∶1000, R&D Systems) and HRP-linked rabbit anti-goat secondary antibody (1∶5000, Dako, Cambridge, UK), diluted in 5% non-fat milk (Marvel, Lincs UK). Membranes were washed in TBST and the immune complexes visualised by chemiluminescence using the ECL detection kit and an ECL film-based technique (GE Healthcare, Amersham, UK). Equal loading of protein was confirmed by stripping the blot in Restore Western stripping buffer (Pierce, Rockford, USA) for 30 minutes at 37°C and subsequent re-probing with HRP-conjugated anti β-actin antibody (1∶25000, Sigma) for 60 minutes. Densitometric analysis was performed using Quantity One software (Bio-rad, UK).

### Measurement of Collagen Production

After fixation in 4% PFA, cell layers were stained with ‘Sircol’ dye reagent (Sirius red and picric acid, Biocolor Ltd, County Antrim, UK) for 1 hour at room temperature. Unbound dye was removed with 0.001 M HCl, the matrix-bound stain solubilised in 0.1 M NaOH and absorbance measured at 570 nm.

### Statistical Analysis

Data are expressed as the mean ± SEM of at least 3 experiments. Statistical analysis was performed by one way analysis of variance (ANOVA) – general linear model. P<0.05 was considered to be significant and noted ‘*’; P values of <0.01 and <0.001 were noted as ‘**’ and ‘***’ respectively.

## Results

### Matrix mineralization and E11 Expression in MLO-A5 cell cultures

**Figure 1:**
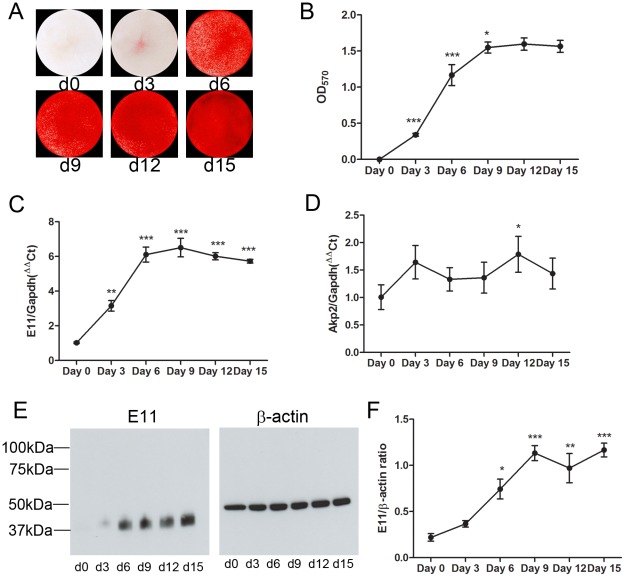
Mineralization and E11 expression by the MLO-A5 cell line. (A) Alizarin red staining of MLO-A5 cultures over a 15-day time-course. (B) Quantification of Alizarin red staining by spectrophotometry. (C) *E11* mRNA expression in the MLO-A5 cultures as determined by qRT-PCR. (D) *Akp2* mRNA expression in MLO-A5 cultures. (E) E11 protein (∼38 kDa) expression by MLO-A5 cells. β-actin was used as a loading control. (F) Densitometric analysis of 3 independent E11 western blots. Results are mean±SEM (n = 3). * P<0.05, ** P<0.01, *** P<0.001 compared with previous time-point (B) or day 0 (C and F).

Alizarin red staining was initially evident on day 3 of culture (Figures 1A and B), increased on days 6 and 9, but showed no further increases thereafter. These studies are in agreement with previously published data [27] and confirm that mineralization of the MLO-A5 cell cultures was initiated by day 3 and almost complete by day 9.

**Figure 2:**
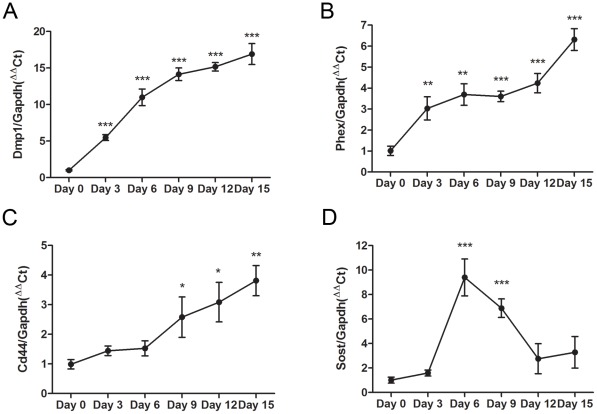
Osteocytic differentiation of the MLO-A5 cell line. The temporal expression patterns of the osteocyte-marker genes *Dmp1* (A), *Phex* (B), *Cd44* (C) and *Sost* (D) as assessed by qRT-PCR. Results are mean±SEM (n = 3). * P<0.05, ** P<0.01, *** P<0.001 compared to day 0.

**Figure 3:**
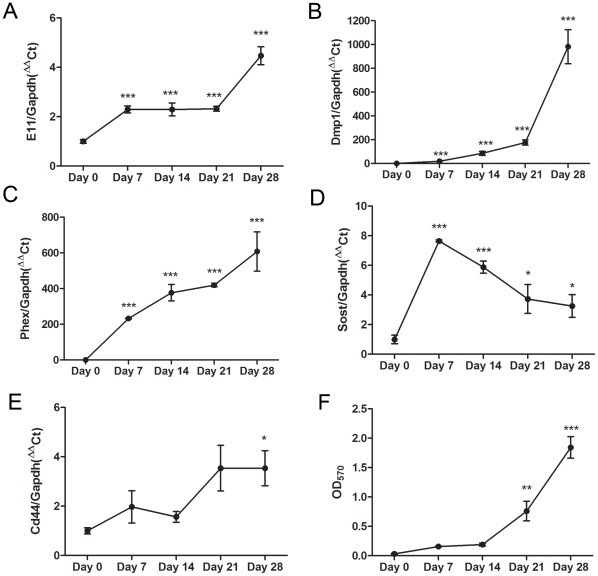
Osteocytic differentiation of primary calvarial osteoblasts *in vitro*. The temporal expression patterns of the osteocyte marker genes, *E11* (A), *Dmp1* (B), *Phex* (C), *Sost* (D) and *Cd44* (E) as assessed by qRT-PCR over 28 days of culture. (F) Mineralization of the primary osteoblast cultures as assessed by Alizarin red staining. Results are mean±SEM (n = 4). * P<0.05, ** P<0.01, *** P<0.001 compared to day 0.

*E11* mRNA levels were detectable at day 0 of MLO-A5 cell culture, increasing concomitantly with matrix mineralization at days 3, 6 and 9, at which time *E11* mRNA expression levels reached an apparent plateau (Figure 1C). A similar temporal pattern of expression was also observed for E11 protein levels (Figure 1E and F), although E11 protein expression was not observed at day 0 of culture. In contrast, although the MLO-A5 cells were found to express mRNA for tissue non-specific alkaline phosphatase (*Akp2*), as previously observed by Kato *et al.* [27], no obvious change in the temporal expression pattern of*Akp2* mRNA was observed during the entire 15-day culture period (Figure 1D). Increases in MLO-A5 cell expression of *Dmp1*, *Phex*, *Sost* and *Cd44* mRNA was also noted during acquisition of an osteocyte-like phenotype by MLO-A5 cells at more advanced stages of mineralization (Figure 2). This temporal profile in gene expression was also observed in mineralizing primary calvarial osteoblasts (Figure 3) confirming the suitability of the MLO-A5 cell line as a model to study osteocytogenesis. Together these results indicate that under mineralizing conditions MLO-A5 cells differentiate into an E11-expressing osteocyte-like phenotype.

### ECM mineralization is required for E11 expression

**Figure 4:**
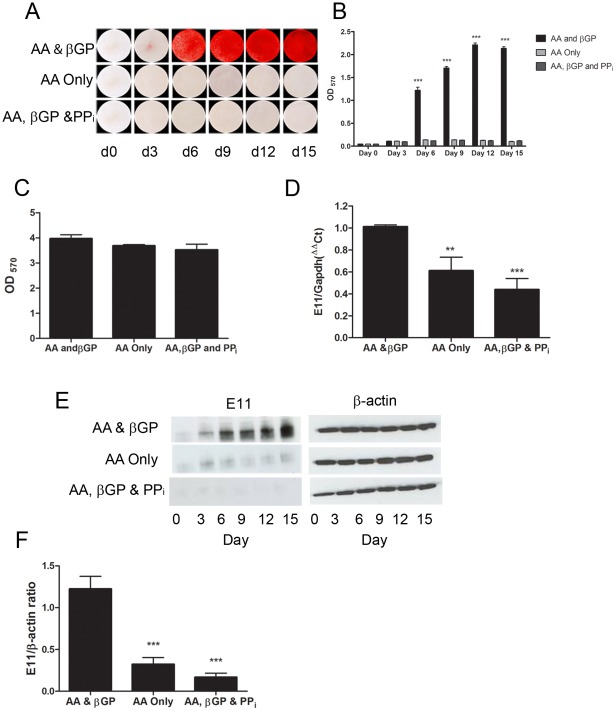
Inhibition of matrix mineralization in the MLO-A5 cell-line. (A) Alizarin red staining of MLO-A5 cultures in which mineralization was inhibited by the addition of PP_i_ or the omission of βGP and quantified by spectrometry (B). (C) Quantification of the collagenous matrix produced by the MLO-A5 cell after 9 days of culture as demonstrated by Sirius red staining (D) *E11* mRNA expression in the mineralization-inhibited MLO-A5 cell cultures. (E) E11 protein expression in mineralization-inhibited and control MLO-A5 cultures as demonstrated by western blotting (F) Densitometric analysis of the protein expression at day 9 in 3 independent E11 western blots. Results are mean±SEM (n = 3). ** P<0.01, *** P<0.001 compared to day 0 (B) or AA & βGP (D and F) cultures.

To further explore the link between E11 expression and ECM mineralization first proposed by Irie *et al.* [25], we constrained *in vitro* mineralization by two different approaches; by either adding PP_i_, the natural mineralization inhibitor, or by omitting βGP, the phosphate donor from the culture medium. Predictably, both approaches resulted in marked ablation of mineralization in the MLO-A5 cell cultures, with a greater than 15-fold (P<0.001) suppression in mineral deposition (Figures 4A and B). Under these PP_i_-supplemented conditions or in the absence of βGP, we found that E11 protein expression levels in MLO-A5 cells were significantly reduced compared to control cultures. This reduction in E11 protein expression was more marked in the PP_i_ supplemented cultures (Figures 4E and F). *E11* mRNA expression levels were also significantly reduced in the absence of mineralization achieved by either of these two approaches (Figure 4D). Intriguingly, however, *E11* mRNA expression was observed in MLO-A5 cells even in cultures where mineralization was completely absent; thus, qRT-PCR Ct values of 26–27 were evident despite negligible E11 protein and mineralization. These findings suggest that *E11* mRNA is still expressed in the mineralization-inhibited cultures, even though the protein is not (Figures 4E and F), and is consistent with the time course of E11 mRNA and protein expression shown in Figures 1C and E. Measurement of matrix-bound deposition of collagen showed that the inhibition of matrix mineralization induced by either omission of βGP or supplementation with PP_i_ was not associated with any marked impairment of collagen synthesis, which was unaffected under either of these conditions (Figure 4C).

**Figure 5:**
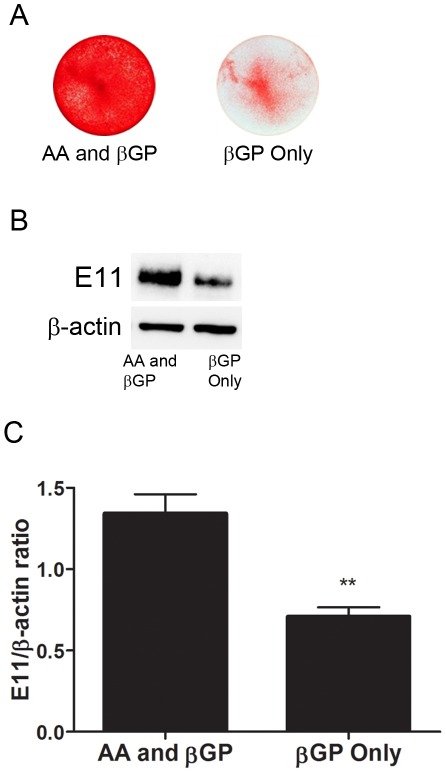
Effect of inorganic phosphate supplementation on MLO-A5 cultures. Matrix mineralization (A) and E11 protein expression (B) after 9 days in control (AA and βGP) MLO-A5 cultures and cultures in which AA was omitted (βGP only). (C) Densitometric analysis of 3 independent E11 western blots. Results are mean±SEM (n = 3). ** P<0.01 compared to AA & βGP cultures.

To investigate whether the presence of P_i_ alone is sufficient to drive E11 expression, MLO-A5 cells were cultured in the presence of AA and βGP (control) or with βGP alone. As expected, the cultures supplemented with βGP alone demonstrated reduced mineral deposition compared to control cultures, although some evidence of dystrophic mineralization was present, even in the absence of ascorbic acid-induced collagen secretion (Figure 5A). Limited E11 protein expression was also observed in the cultures supplemented with only βGP (Figure 5B and 5C) but this was significantly less than the amount of E11 expressed by the cultures supplemented with AA and βGP. This suggests that the deposition of mineral within the ECM is required to fully drive E11 expression and osteocyte differentiation.

### ECM mineralization is required for osteoblast-to-osteocyte transition

**Figure 6:**
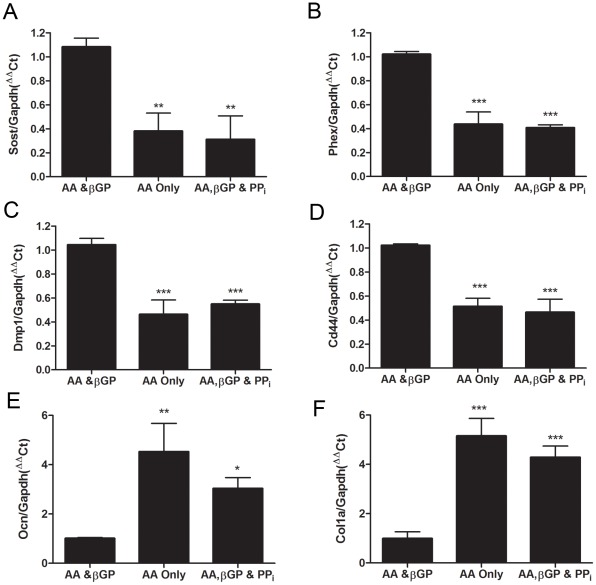
Effect of ECM mineralization inhibition on the expression of osteocyte and osteoblast marker genes in MLO-A5 cells. *Sost* (A), *Phex* (B), *Dmp1* (C), *Cd44* (D), *Ocn* (E) and *Col1a* (F) mRNA expression in the mineralization-inhibited cultures as quantified by RT-PCR. Results are mean±SEM (n = 3). * P<0.05, ** P<0.01, *** P<0.001 compared to AA & βGP cultures.

We have shown that the expression levels of several osteocyte marker genes are increased during osteocytic differentiation and matrix mineralization by MLO-A5 cells *in vitro* (Figure 2). To confirm whether inhibition of mineralization (by adding PP_i_ or omitting βGP) also negatively regulates expression of these genes, as had been shown for *E11* mRNA levels, we measured the expression of a panel of osteocyte marker genes (*Sost*, *Phex*, *Dmp1* and *Cd44*) by RT-qPCR after 9 days of culture. We found that the levels of all of these genes was decreased in cultures either lacking βGP, or supplemented with PP_i_, compared to control cultures supplemented with both AA and βGP (Figures 6 A–D). Decreased level of osteocyte marker genes in these MLO-A5 cells suggests that matrix mineralization is required for the transition from a mature osteoblast to an osteocyte-like phenotype. The likelihood that the lack of mineralization is sufficient to block this osteocytogenesis *in vitro* was confirmed by analyzing osteoblast-marker gene expression, which showed that the levels of osteocalcin (*Ocn*) and collagen type I alpha (*Col1a*) mRNA were both elevated in these cultures where mineralization had been inhibited by either exogenous PP_i_ or by omission of βGP (Figures 6E and F).

### Recovery of in vitro mineralization by MLO-A5 cells

**Figure 7:**
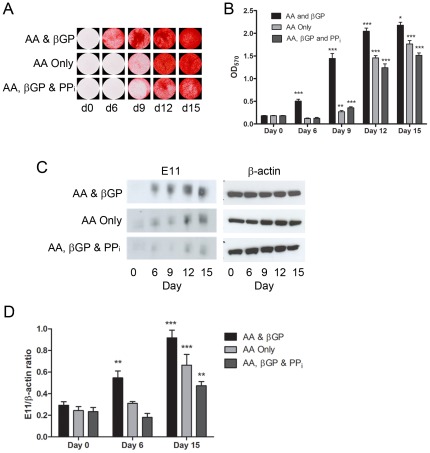
Effect of reversal of ECM mineralization inhibition on MLO-A5 cells. (A) Alizarin red staining of MLO-A5 cultures in which mineralization was inhibited for 6 days before being promoted for a further 9 days. (B) Quantification of the results from (A) by spectrometry. Results are mean±SEM (n = 3). (C) E11 protein expression under the same conditions as (A) and (B). (D) Densitometric analysis of 3 independent E11 western blots. Results are mean±SEM (n = 3). *P<0.05, **P<0.01, ***P<0.001 compared to previous time-point.

It is possible that MLO-A5 cells undergo some irreversible change in cell behaviour in response to inhibition of mineralization. If this were indeed the case, then it would be possible that the suppression of E11 expression may not be directly linked to mineralization status. We therefore determined whether MLO-A5 cells were capable of recovering both their mineralization potential and E11 expression after periods of inhibited matrix mineralization. As expected, Alizarin red staining was completely lacking after treatment for 6 days with PP_i_ or culture in the absence of βGP (Figure 7A). In contrast, some albeit limited mineralization was seen by day 9, 3 days after the cessation of inhibition upon return to culture conditions that promote differentiation. Evidence for such recovery in mineralization increased with more prolonged culture, but yet failed to reach control levels even at days 12 or 15 (Figure 7B). Furthermore, the link between modified mineralization status and E11 expression was strengthened by the observations that such re-initiation of mineralization was intimately associated with increased E11 expression (Figures 7C and D). These data further strengthen the connection between the mineralization of MLO-A5 cell cultures and the expression of E11.

## Discussion

The density, integrity and three-dimensional organisation of the osteocyte canalicular network are important in the etiology of various skeletal disorders such as osteoporosis and osteomalacia [30]. Such changes suggest a cellular basis for many bone diseases and it is therefore essential that we understand more fully the processes and genetic circuitry responsible for the terminal differentiation of osteoblasts to osteocytes. Although, the mechanisms which govern these osteocytogenic processes are unknown, several models of osteocyte differentiation have been proposed [3]–[5], [31]. Such models, applicable to both intramembranous and endochondral ossification, have proposed that the osteocyte has a predominantly passive role [31], [32]. Other evidence, however, has suggested that the embedding of an osteocyte is an active, invasive process [6]. Indeed, it is possible to speculate that the dramatic morphological and genetic transformations observed during osteocytogenesis, reflect a tightly regulated, active process that does not merely involve the osteoblast simply becoming trapped, without an inherent contribution, within the ECM.

The role of the mineralized ECM in the regulation of osteocyte development is now becoming more fully recognised. Mineralization of lamellar bone occurs at the late osteoblast/pre-osteocyte stage and it has been suggested that ECM mineralization is part of the process that transforms pre-osteocytes into mature osteocytes [17]. This is supported by data showing a positive correlation between ECM mineralization and osteocyte number [8], [33]–[35]. These *in vivo*-based observational studies cannot, however, entirely rule out the possibility that defective osteocyte maturation/morphology may lead to changes in bone mineralization. For this reason, the present study has exploited a carefully defined, controlled *in vitro* model system in which the MLO-A5 cell-line effectively models late-stage osteoblastic differentiation to investigate whether mineralization of the surrounding ECM acts as a trigger for osteocyte maturation.

Inhibition of ECM mineralization using two distinct strategies resulted in a striking impairment of E11 protein expression. These impaired E11 levels were apparent even at late stages of the 15-day long culture period, when strong E11 protein expression would normally have been be expected. It was apparent that inhibition of mineralization using PP_i_ also resulted in a marked suppression of E11 expression, greater than achieved by the omission of βGP alone, therefore suggesting that presence of PP_i_ inhibits E11 protein expression. It has previously been shown that E11 expression is required for the elongation of osteocyte cellular processes [15] and that mineralization occurs along the processes produced by differentiating MLO-A5 cells [17] and primary osteoblasts [33], [36]. Our studies strengthen this link between E11-mediated process formation and ECM mineralization. The relative lack of E11 protein in cultures in which mineralization has been inhibited is suggestive of a block in MLO-A5 differentiation toward the osteocyte phenotype. This was confirmed by the decreased expression of the osteocyte-marker genes *Dmp1*, *Phex*, *Sost* and *Cd44* by MLO-A5 under mineralization-inhibited conditions; conversely, the expression of the osteoblast-marker genes *Col1α* and *Ocn* was increased. Taken together, these data strongly suggest that inhibition of mineralization promotes retention of a late-osteoblast stage of differentiation and prevents osteocytic differentiation. An unexpected observation was the decreased expression of Sost, while E11, DMP1 and Phex increase, during mineralisation in both primary and MLO-A5 cell cultures. This is currently unexplained but its examination may give clues to the mechanism by which Sost expression is regulated. Future studies may also broaden this strategy to include bone markers known to regulate ECM mineralization, such as other SIBLINGs (BSP, OPN) and OCN.

The mechanism by which ECM mineralization promotes osteocytic differentiation is presently unknown. The osteocyte has been shown, however, to be highly responsive to changes in its mechanical environment [1], [37]–[39]. Indeed, primary cilia described on both osteoblasts and osteocytes [40] are known to play important roles in transducing external mechanical cues, derived either directly or indirectly from the ECM, into intracellular responses essential for processes such as differentiation [40]–[42]. It has also been reported that subjecting pre-osteoblasts and osteoblasts to hypoxic conditions *in vitro* leads to increased expression of osteocyte-markers [43]. It seems reasonable to assume therefore that mineralized matrices, which are more hypoxic than non-mineralized osteoid, may positively influence osteocytogenesis *in vivo*. Maintenance of MLO-A5 cells in hypoxia resulted in decreased mineralization compared to cells cultured under normoxic conditions, however, indicating that this effect may also depend on the stage of differentiation [44].

Interestingly, increased E11 protein was observed in osteocytes from the DMP1-null mouse, which is characterized by diffuse osteomalacia and reduced mineral density [13]. It is important to note, however, that although the bone from these mice is hypomineralized, mineral is still present within the matrix and could potentially drive E11 expression. Indeed, in wild-type mice, E11 protein expression in cortical bone is localized to the immature osteocytes within newly-mineralizing osteoid [15]. Such newly-synthesized bone would be expected to have a lower mineral density than the more mature bone, similar to the hypomineralized bone of the DMP1-null mouse. Therefore, the gradient of mineral density and matrix maturation may regulate E11 expression. Also, in the DMP1-null mouse, other factors such as increased alkaline phosphatase and FGF23 levels may affect E11 expression independent of ECM mineralization.

A recent study by Zhang *et al.* [45] found that restoration of serum P_i_ levels in DMP1-null mice, partially rescued the negative effect on osteocyte differentiation observed in this mouse model. This was also accompanied by a decrease in osteoid volume, suggesting partial restoration of mineralization. However, discriminating if the presence of P_i_ alone is sufficient to drive osteocytogenesis, or whether its incorporation into the ECM as hydroxyapatite is required for osteocyte maturation, has not been fully explored. Our results using βGP as a source of phosphate suggest that its presence alone, without a collagenous matrix, can induce limited expression of the osteocyte marker E11. However, this level of expression is significantly less that that observed when the cells are also cultured with AA, enabling the synthesis and secretion of a collagenous ECM. Therefore, both the bio-availability of P_i_ and its deposition within the ECM appear to be essential for osteocyte differentiation.

E11 has previously been described as the earliest osteocyte marker during osteoblast-osteocyte transition [15], [17]. Our studies indicate that E11 is also intimately regulated by the ECM mineralization status. It is important to note, however, that these studies have taken place in a 2D model system which may not be fully representative of the *in vivo* bone environment, and that our attempts to test this through shRNA-mediated knockdown in MLO-A5 cells have not been successful in blocking E11 expression. Although further studies are required to better define the mechanisms that control transcription, translation and post-translational stability of the E11 protein, these results emphasise the influence of physiological ECM-cell interactions on osteoblast differentiation and importantly suggest that ECM mineralization is essential for terminal osteoblast differentiation to the osteocyte phenotype.
